# Radiation-induced morphea—a rare but severe late effect of adjuvant breast irradiation: Case report and review of the literature

**DOI:** 10.1007/s00066-018-1336-9

**Published:** 2018-07-16

**Authors:** Richard Partl, Peter Regitnig, Gerlinde Tauber, Michaela Pötscher, Vesna Bjelic-Radisic, Karin S. Kapp

**Affiliations:** 1 Department of Therapeutic Radiology and Oncology, Comprehensive Cancer Center (CCC), Medical University of Graz, Auenbruggerplatz 32, 8036 Graz, Austria; 2 Institute of Pathology, Comprehensive Cancer Center (CCC), Medical University of Graz, Auenbruggerplatz 25, 8036 Graz, Austria; 3 Division of Gynecology, Comprehensive Cancer Center (CCC), Medical University of Graz, Auenbruggerplatz 14, 8036 Graz, Austria

**Keywords:** Morphea, Radiotherapy, Scleroderma, localized, Side-effect, Breast cancer, Morphea, Bestrahlung, Lokalisierte Sklerodermie, Nebenwirkung, Brustkrebs

## Abstract

**Background:**

Radiation-induced morphea (RIM) is a circumscribed localized scleroderma that occurs most often in the breast. After an asymptomatic period of one month to several years, the symptoms (circumscribed inflammation, edema, sclerosis) often arise suddenly and cannot be clinically distinguished from a local recurrence in the form of inflammatory carcinoma.

**Case:**

We present a case of a 74-year-old woman who developed this rare and serious local side-effect in connective tissue following neoadjuvant CDK 4/6 inhibitor abemaciclib (Verzenio®) and aromatase inhibitor anastrozole (Arimidex®) therapy and subsequent radiation therapy of the breast.

**Conclusions:**

Little is known about risk factors and pathogenesis of RIM. Here we describe the first case of RIM following immunotherapy. The diagnosis is based on clinical appearance and histopathological examination. Treatment should be initiated in the inflammatory stage in order to prevent or delay irreversible fibrosis and atrophy of the breast.

## Zusammenfassung

**Hintergrund:**

Die strahleninduzierte Morphea (RIM) ist eine lokale begrenzte Sklerodermie mit bevorzugter Lokalisation in der Brust. Nach einem beschwerdefreien Intervall von einem Monat bis mehreren Jahren treten die Symptome (umschriebene Entzündung, Ödem, Sklerose) häufig abrupt auf und sind klinisch nicht von einem Lokalrezidiv im Sinne eines inflammatorischen Karzinoms zu unterscheiden.

**Fallbericht:**

Wir berichten den Fall einer 74-jährigen Patientin, die nach neoadjuvanter Behandlung mit dem CDK 4/6 Inhibitor Abemaciclib (Verzenio®) und dem Aromataseinhibitor Anastrozol (Arimidex®) sowie adjuvanter Radiotherapie der Brust diese schwere lokale Nebenwirkung des Bindegewebes entwickelte.

**Schlussfolgerung:**

Risikofaktoren für die Entstehung einer RIM sowie deren Pathogenese sind noch weitgehend unklar. Wir beschreiben den ersten Fall einer RIM nach Immuntherapie. Die Diagnose wird anhand des klinischen Verlaufs und der histologischen Untersuchung gestellt. Die Behandlung sollte bereits im inflammatorischen Stadium eingeleitet werden, um eine irreversible Fibrose und Atrophie der Brust abzuwenden oder zu verzögern.

## Introduction

Reversible acute skin changes within the radiation field (NTC grade 1 or 2) are common side-effects induced by radiation of the breast with an incidence of >90% [1]. Irreversible late effects (telangiectases, subcutaneous indurations, liponecrosis, fibrosis) are rare in connection with modern irradiation techniques. Radiation-induced morphea (RIM) is distinct from these and is a rare and often unrecognized local and chronically progressive radiation-associated scleroderma of the skin [2, 3]. Clinical and surgical oncologists should bear this condition in mind because only a rapid diagnosis and treatment of RIM can stop or delay the progress of irreversible fibrosis of the cutis and subcutis and thus improve the quality of life for the patients. Here we report the case of a patient who developed an early and extensive RIM of the breast following therapy with neoadjuvant cyclin-dependent kinase (CDK) 4/6 inhibitor and aromatase inhibitor (anastrozole, Arimidex®), segmental resection and adjuvant radiotherapy.

## Case presentation, clinical follow-up, and examination findings

In October 2015, in the course of a routine mammography and sonography, a 72-year-old woman was diagnosed with a centrally located carcinoma of the right breast with enlarged axillary lymph nodes. The pretherapeutic staging tests and anamnesis were unremarkable apart from hypertension, obesity and smoking and there was no history of allergy. In particular, there were no signs of an autoimmune disease.

Based on the clinically positive axilla of a cT1 tumor (invasive carcinoma of no special type, G1, hormone receptor positive, Her2/neu negative, Ki67 10%), the patient was given a 4-month neoadjuvant systemic therapy with the nonsteroidal aromatase inhibitor anastrozole (Arimidex®) and the CDK 4/6 inhibitor abemaciclib (Verzenio®), from November 2015 to March 2016, as part of a clinical trial (NeoMONARCH).

**Fig. 1:**
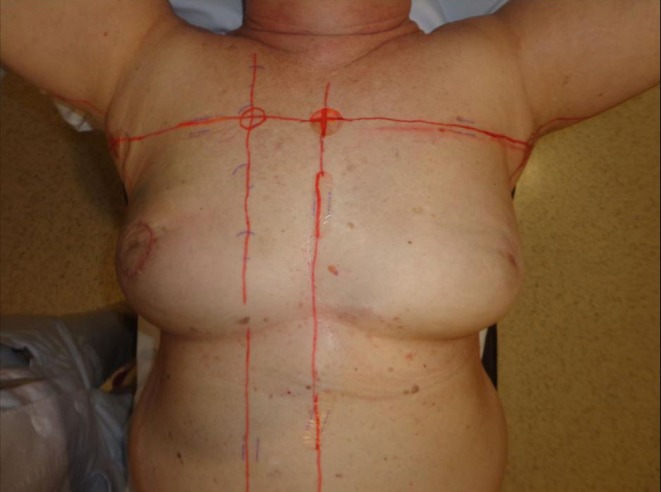
Prior to radiotherapy the volume of the irradiated right breast revealed no difference compared with the left side

The histopathological work-up of the surgical specimen revealed stage ypT1b ypN0 R0 disease. Following segmentectomy and sentinel node dissection, adjuvant radiotherapy (RTX) of the right breast and the supraclavicular region was done in three-dimensional (3D) conformal technique up to a total dose of 50 Gy (6MV) in 25 fractions with an electron boost dosage to the tumor bed of 10 Gy (16 MeV) in 5 fractions while continuing therapy with anastrozole. Prior to radiotherapy the measured volume of the irradiated right breast revealed no difference compared with the left side (1455 vs. 1500 ccm; Fig. 1). Towards the end of the course of radiation, the patient developed a moderate acute radiodermatitis with small circumscribed moist epitheliolysis in the submammary fold, which were classified as CTCAE grade 2 and treated symptomatically for the remaining period of radiotherapy.

**Fig. 2:**
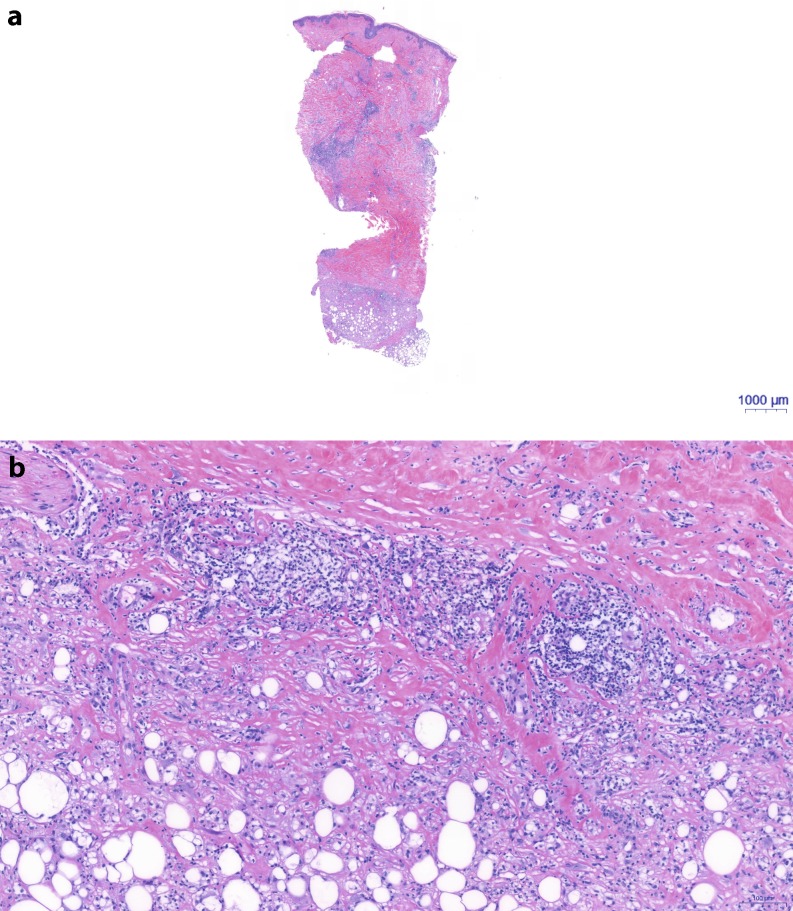
**a** Haematoxylin and eosin stained section of the deep punch biopsy showing massive fibrosis of dermis (*red* stained areas) and pronounced perivascular and subcutaneous inflammatory infiltrate (*blue* stained areas). **b** Magnification of Fig. 2**a** at the interphase between dermis and subcutis: Lymphoid infiltrate on dermal site and histocytoid infiltrate towards adipocytes with consumption of adipocytes and increase of collagen. At the bottom loosely cohesive collagen and towards the dermis increasing thick and dense collagen bundles

Three months after completion of RTX, all acute skin changes had completely healed, but a new, 2 cm wide, circumscribed cutis edema was observed at 1 o’clock within the irradiated breast and was documented. Six months after RTX, this developed into an increasing local redness and induration of the skin. A cutis edema was visible in sonography, which showed that the changes were limited to the irradiation field. Nine months after RTX, the cutis edema had grown to cover the whole former irradiation field, the skin exhibited a continuous inflammatory infiltrate, hyperpigmentation and induration. A loss of breast volume was also clearly evident. To rule out a lymphangiosis carcinomatosa cutis (inflammatory carcinoma) recurrence, a targeted punch biopsy was performed. The histology showed no signs of malignant tumor cells but a pronounced dermal fibrosis with thickened dermis and fibrosis extending into the underlying fatty tissue, with corresponding panniculitis and pronounced chronic perivascular inflammation (Fig. 2a, b).

**Fig. 3:**
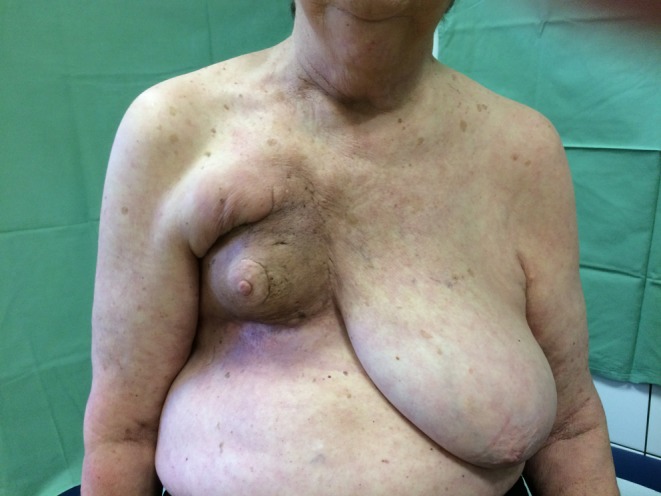
A significant loss of volume, induration and hyperpigmentation was evident 20 months after completion of radiotherapy

Based on the pronounced clinical picture (Fig. 3) and the distressing situation for the patient, the histological findings from February 2017 were re-examined. Taking account of the radiation history and the clinical progression, a postradiogenic circumscribed scleroderma (morphea) was diagnosed in December 2017, 20 months after RTX.

After the diagnosis had been established all suggested treatments which included systemic immune suppression with steroids and methotrexate (MTX) were declined by our patient. She has been applying topical steroids and has undergone several weeks of lymph drainage at a specialized center. The clinical picture has remained unchanged since December 2017.

## Discussion and literature review

Radiation-induced morphea (RIM), also known in the literature under the names postirradiation morphea (PIM), radiation-induced scleroderma, radiation port morphea, radiation port scleroderma, localized scleroderma and circumscribed scleroderma, is a chronic inflammatory condition of the skin and underlying tissue which results in a fibrotic transformation and in very rare cases may involve fascia and bone. Bleasel et al. [2] and Davis et al. [4] each describe frequencies of 1:500 irradiated breast cancer patients. In the nonirradiated population, an incidence of 2.7:100,000 persons per year has been reported [5]. The difference in incidence strongly suggests that radiotherapy is a risk factor. However, since 1989 [6] only 81 cases of RIM have been described in the literature. If the reported frequency of 1:500 irradiated breast cancer patients holds true, a significant number of patients developing RIM have gone undiagnosed or misdiagnosed and may have received no or inappropriate treatment. In a recent report, Friedman et al. [7] described 3 cases of RIM in 12,000 breast cancer patients treated with adjuvant radiotherapy resulting in an estimating prevalence of 1:3000 cases. The onset of RIM in these patients was 4, 5, and 7 years, respectively.

Of note is the fact that the large majority of reports relate to adjuvant radiotherapy of the breast. The numbers of cases reported in connection with head and neck cancer [8], endometrial carcinoma [9], vulva [10] or lymphomas [11] are significantly smaller. A pragmatic explanation might be the ease of clinical diagnosis and of the comparison between the irradiated and nonirradiated breast [12]. Another theory suggests that the cause is the inclusion of substantial dermal and subdermal tissue in the irradiated volume [13]. Table 1 summarizes the cases of RIM reported after adjuvant irradiation of the breast over the last 20 years.

**Table 1:** Published reports on localized morphea after adjuvant irradiation of the breast since 1998

Author	No. of cases	Time interval between radiotherapy and onset	Prior systemic treatment
Gollob et al. (1998) [29]	1	1 < year	Not recorded
Bleasel et al. (1999) [2]	4	1 < year	2 cases: tamoxifen; 2 cases: no systemic treatment
Fischer et al. (1999) [25]	1	14 years	Not recorded
Schaffer et al. (2000) [20]	2	6.5–32 years	1 case: tamoxifen; 1 case: not recorded
Ullen and Björkholm (2003) [9]	1	2 months	Not recorded
Ardern-Jones and Black (2003) [30]	1	13 years	Tamoxifen
Reddy et al. (2005) [31]	1	<1 year	Tamoxifen
Dubner et al. (2006) [32]	1	3 years	Chemotherapy (not specified)
Dancey and Waters (2006) [33]	1	<1 year	Not recorded
Seale et al. (2008) [34]	1	2 years	Doxorubicin, cyclophosphamide
Walsh et al. (2008) [35]	5	4–12 years	1 case: antiestrogen treatment; 4 cases: no treatment
Cheah et al. (2008) [36]	1	9 months	Tamoxifen
Herrmann et al. (2009) [11]	1	1.5 years	Anti-hormonal therapy
Morganroth et al. (2010) [37]	1	6 years	Doxorubicin, cyclophosphamide, paclitaxel
Laetsch et al. (2011) [38]	3	<1 year	1 case: doxorubicin and cyclophosphamide, tamoxifen; 2 cases: no systemic treatment
Wernicke et al. (2011) [39]	1	1.5 years	Tamoxifen
Alhathlool et al. (2012) [40]	1	2.7 years	Anastrozole
Lim et al. (2014) [41]	1	7 months	Epirubicin, cyclophosphamide, docetaxel
García-Arpa et al. (2015) [42]	1	1 year	Chemotherapy (not specified), letrozole
Yanaba et al. (2015) [43]	1	3 months	Not recorded
Dyer et al. (2016) [44]	2	3–4 months	1 case: chemotherapy (not specified)
Chu et al. (2017) [45]	1	10 months	Not recorded
Gonzalez-Ericsson et al. (2018) [46]	1	1.3 years	Cisplatin, paclitaxel
Friedman et al. (2018) [7]	3	4, 5, 7 years	Case 1: neoadjuvant chemotherapy (not specified), adjuvant tamoxifen; case 2: adjuvant tamoxifen; case 3: non
Peterson et al. (2018) [23]	1	5 months	Not recorded
Papanikolaou et al. (2018) [27]	1	4 months	Not recorded
Partl et al. (current report)	1	3 months	Anastrozole, CDK4/6 inhibitor (abemaciclib)

Currently no predictive model for the risk of developing RIM exists. There is no relationship with the radiation parameters such as total dose and single dose [2], with acute radiation side effects, age, neoadjuvant or concomitant systemic cancer therapy [14]. In patients with systemic sclerosis there is no evident difference with respect to acute skin toxicity, which are known to carry a significantly higher risk of developing chronic side-effects compared to the control group (29.1% vs. 14%; *p* = 0.001) [15, 16]. This means that the decision on whether to use RTX needs to be made carefully and that the risk should be discussed with all patients diagnosed with systemic sclerosis.

Our patient received neoadjuvant treatment with the CDK4/6 inhibitor abemaciclib (Verzenio®). CDK 4 and 6 regulate the transition from the G1 to S phase through the inhibition of the tumor suppressor function of the retinoblastoma protein. The novel cancer therapeutic abemaciclib is a highly selective reversible inhibitor of these enzymes and received FDA Breakthrough Therapy designation in October 2015. Its cell cycle inhibition is based on the liberation of the tumor suppressor retinoblastoma protein from the inhibitory effect of the cyclin-dependent kinase [17, 18]. At present it is unclear whether the pathology described in our case report is related to the neoadjuvant CDK 4/6 inhibitor. Nonomura et al. however postulated that cyclin-dependent kinase 4/6 proteins can modulate the production of inflammatory molecules through multiple pathways in patients with rheumatoid arthritis [19]. The fact that interactions of the newest generation of medicines have not been tested prospectively makes it even more important to record and report such side-effects that arise in combination with radiotherapy.

Usually the symptoms of RIM manifest within a year after the end of radiotherapy, but both short and very long intervals, from one month to 32 years, have been described [20].

Typically, following a variable period of asymptomatic latency, there is an abrupt onset of edematous and erythematous plaques (initial inflammatory phase). The subsequent sclerotic phase is mainly characterized by painful induration of the irradiated breast, followed by fibrotic retraction and hyperpigmentation. The changes are usually limited to the area of the irradiated area but in rare cases can extend beyond this area [3, 21] or even become generalized [22]. Peterson et al. presented an unusual overlap of morphea and lichen sclerosus. Both skin disorders are considered inflammatory autoimmune phenomena favoring distinct tissue planes [23].

Diagnosis is done by biopsy. Histologically, in the inflammatory phase dermal perivascular and interstitial inflammatory infiltrates are found. In the sclerosing phase a sclerotic reorganization of the tissue due to an increase of collagen occurs. The epidermis remains uninvolved. In the inflammatory phase the differential diagnosis must consider an infection, a “radiation recall reaction” and an inflammatory tumor recurrence. In the “burn-out phase” a chronic radiodermatitis and a related postradiogenic fibrosis are possible.

The pathomechanism for the development of RIM is not fully understood. It is hypothesized to be a disorder of immune regulation against the background of a genetic predisposition. A trigger (e. g., irradiation, infection, trauma) activates expression of cytokines (IL 4, 5) and transforming growth factor-β (TGF-β), which leads to an activation of fibroblasts and an increase in collagen synthesis [4, 11]. TGF-β induces an excessive transformation of CD34-positive fibroblast precursor cells into myofibroblasts. This in turn leads to a thickening and sclerosis of the connective tissue. Through a positive feedback mechanism, TGF-β stimulates its own synthesis [24].

Treatment depends on the stage of the inflammation. In the acute inflammation phase immunosuppressive drugs are recommended. As a first-line therapy, topical application of calcineurine inhibitors (tacrolimus ointment) and topical steroids are recommended. Systemic immune suppression with steroids, MTX und cyclosporine can also be used. For symptomatic improvement of the fibrosis, some authors recommend local application of heparin, hyaluronidase [25], ultraviolet A irradiation [26] or penicillin (3 × 10^6^ IU daily for two weeks). In another case report photodynamic therapy is suggested as a successful treatment option [27]. Testing of the different therapy options in the largest RIM cohorts to date showed the best response to systemic treatment with MTX or ultraviolet-B phototherapy [28].

Treatment should begin immediately after diagnosis in the inflammatory stage in order to prevent or delay irreversible fibrosis and atrophy.

## Conclusions

RIM is a rarely described, serious and unpredictable late side-effect with a large variability in the timing of onset. Practitioners in oncology should consider this diagnosis early and should carry out appropriate tests to exclude infection, an inflammatory recurrence of cancer, a radiation recall phenomena, postradiogenic fibrosis or chronic radiodermatitis. After histological confirmation of RIM, it is important to begin local and systemic therapy as soon as possible in order to limit the progress of fibrosis and atrophy and to improve the patient’s quality of life.
